# Supplementary material for: FERN – a Java framework for stochastic simulation and evaluation of reaction networks
Source: BMC Bioinformatics. 2008 Aug 29;9:356. doi: 10.1186/1471-2105-9-356 (PMC2553347; doi:10.1186/1471-2105-9-356)
Supplement: Additional file 1 — FERN distribution, Version 1.3. This archive contains the FERN source code and binaries as well as documentation and example models in FernML and SBML. [file 1471-2105-9-356-S1.zip › fern/doc/slides.pdf]

FERN

Framework for Evaluation of chemical Reaction Networks

Florian Erhard

Institut für Informatik, Ludwig-Maximilians-Universität München

April 15, 2007

Contents

Overview
- Definition
- Example
- Complex?

Contents

Overview
- Definition
- Example
- Complex?

Structure
- Main classes / interfaces
- UML of Network related classes
- Observer system

Contents

Overview
- Definition
- Example
- Complex?

Structure
- Main classes / interfaces
- UML of Network related classes
- Observer system

Additional features
- Gnuplot
- Analysis
- Stochastics

Contents

Overview
- Definition
- Example
- Complex?

Structure
- Main classes / interfaces
- UML of Network related classes
- Observer system

Additional features
- Gnuplot
- Analysis
- Stochastics

Documentation and Examples

Overview

What is FERN?

A fast, extensible and comprehensive framework for simulation and analysis of chemical reaction networks.

Overview

What is FERN?

A fast, extensible and comprehensive framework for simulation and analysis of chemical reaction networks.

What is FERN not?

A second Dizzy / Copasy / Stocks. You cannot do simulations by mouseclick but by a very few lines of code.

Overview

What is FERN?

A fast, extensible and comprehensive framework for simulation and analysis of chemical reaction networks.

What is FERN not?

A second Dizzy / Copasy / Stocks. You cannot do simulations by mouseclick but by a very few lines of code.

Advantage

If you are able to write many reasonable lines of code, you can do arbitrarily complex simulations.

Elementary Example

```
Network net = new RNMLNetwork(new File("some_net.xml"));
Simulator sim = new GibsonBruckSimulator(net);
Observer obs = sim.addObserver(new AmountIntervalObserver(sim,1,X));
sim.start(50);
System.out.println(obs);
```

Complexity

Complicated?

- 100 classes / interfaces
- with 12906 lines

Complexity

Complicated?

- 100 classes / interfaces
- with 12906 lines

But...

- only 3 central classes
- half of the lines are javadoc

FERN

Structure

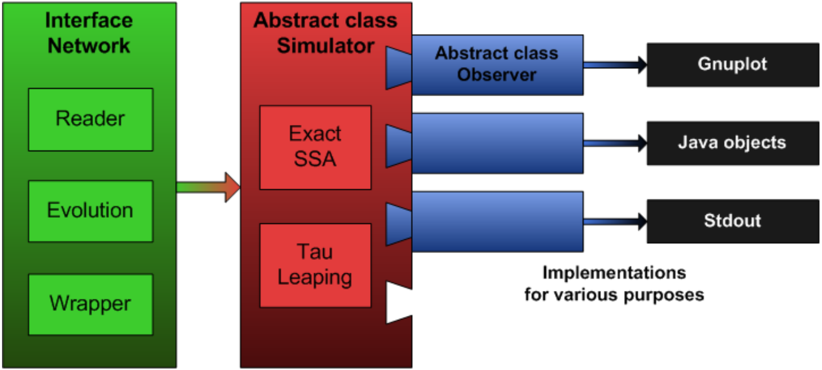
Interface
Network
Reader
Evolution
Wrapper
Abstract class
Simulator
Exact
SSA
Tau
Leaping
Abstract class
Observer
Gnuplot
Java objects
Stdout
Implementations
for various purposes

Structure

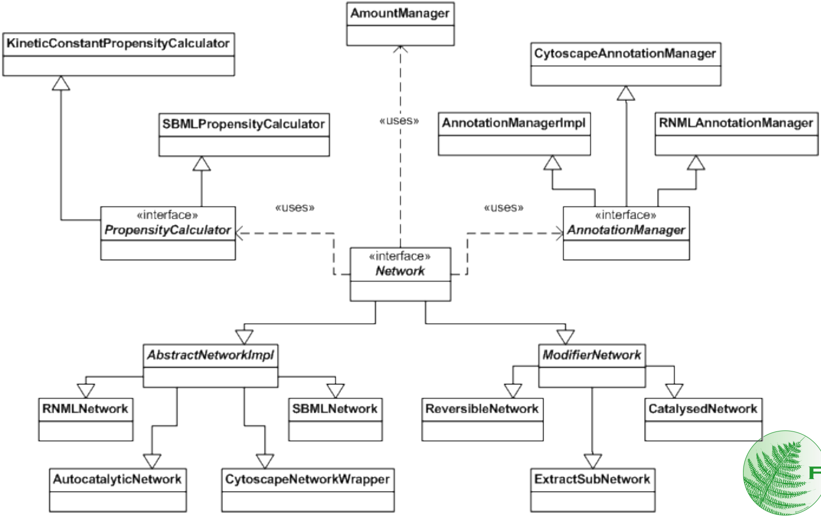
AmountManager
KineticConstantPropensityCalculator
CytoscapeAnnotationManager
SBMLPropensityCalculator
«uses»
AnnotationManagerImpl
RNMLAnnotationManager
«uses»
«uses»
«interface»
PropensityCalculator
«interface»
AnnotationManager
«interface»
Network
AbstractNetworkImpl
ModifierNetwork
RNMLNetwork
SBMLNetwork
ReversibleNetwork
CatalysedNetwork
AutocatalyticNetwork
CytoscapeNetworkWrapper
ExtractSubNetwork

Observer

Observer

Observers can be registered at the Simulator and record various data about the simulation runs. Some are able to handle repeated runs (and yield average data), some are not (refer to the javadoc).

Observer

Observer

Observers can be registered at the Simulator and record various data about the simulation runs. Some are able to handle repeated runs (and yield average data), some are not (refer to the javadoc).

Data

Depending on what kind of data observers record, they provide different kind of data (plain text, gnuplot, histograms,...).

Observer

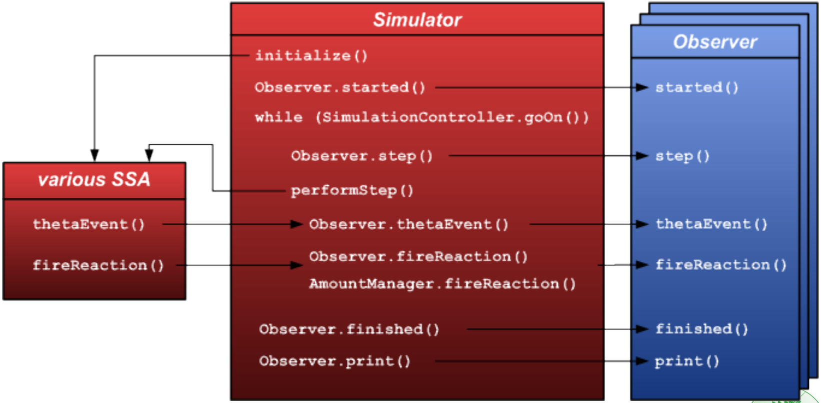
Simulator
initialize()
Observer.started()
while (SimulationController.goOn())
Observer.step()
performStep()
Observer.thetaEvent()
Observer.fireReaction()
AmountManager.fireReaction()
Observer.finished()
Observer.print()
various SSA
thetaEvent()
fireReaction()
Observer
started()
step()
thetaEvent()
fireReaction()
finished()
print()

Gnuplot

A class GnuPlot is included in FERN which enables you to

Gnuplot

A class GnuPlot is included in FERN which enables you to

- retrieve the gnuplot data as String or save it to a file

Gnuplot

A class GnuPlot is included in FERN which enables you to

- retrieve the gnuplot data as String or save it to a file
- get the plot command

Gnuplot

A class GnuPlot is included in FERN which enables you to

- retrieve the gnuplot data as String or save it to a file
- get the plot command
- plot the data by calling gnuplot from java

Gnuplot

A class GnuPlot is included in FERN which enables you to

- retrieve the gnuplot data as String or save it to a file
- get the plot command
- plot the data by calling gnuplot from java
- retrieve the plot as image object or save it to a png file

Gnuplot

A class GnuPlot is included in FERN which enables you to

- retrieve the gnuplot data as String or save it to a file
- get the plot command
- plot the data by calling gnuplot from java
- retrieve the plot as image object or save it to a png file
- show the plot in a JFrame

Analysis

The AnalysisBase class provides

- additional index structures
- generic bfs / dfs

Analysis

The AnalysisBase class provides

- additional index structures
- generic bfs / dfs

Used by included algorithms

- ShortestPaths
- AutocatalyticNetworkDetection

Stochastics

Random number generation

is handled in one central Stochastics class

Stochastics

Random number generation

is handled in one central Stochastics class

- easy replacement of the random number generator

Stochastics

Random number generation

is handled in one central Stochastics class

- easy replacement of the random number generator
- make simulations deterministic by explicitly setting the seed value

Stochastics

Random number generation

is handled in one central Stochastics class

- easy replacement of the random number generator
- make simulations deterministic by explicitly setting the seed value
- count number of random numbers generated from various distributions

Documentation

javadoc

comprehensive documentation of each class and method

Documentation

javadoc

comprehensive documentation of each class and method

Technical overview

A detailed technical overview about FERN's implementation is accessible as pdf document.

Examples

Maybe most important...

There are many examples included in the package fern.example which demonstrate different aspects of FERN.
